# Rare diseases and omics-driven personalized medicine

**DOI:** 10.3325/cmj.2019.60.485

**Published:** 2019-12

**Authors:** Goran Šimić

**Affiliations:** Department of Neuroscience, Croatian Institute for Brain Research, University of Zagreb School of Medicine, Zagreb, Croatia *gsimic@hiim.hr*

This issue of the *Croatian Medical Journal* (*CMJ*) features two articles on rare diseases: one dealing with primary biliary cholangitis (1) and the other with muscular dystrophy and spinal muscular atrophy (SMA) (2). At the *CMJ* editorial board meeting, these articles have generated considerable discussions about problems regarding research and health policies on rare diseases, particularly in a relatively small country such as Croatia.

By definition, a rare disease affects fewer than one individual in 2000 people in the general population. However, as there are nearly 8000 rare diseases (some even without a name), it turns out that these “rare” diseases affect more than 300 million people worldwide (about 6% of the world’s population). Rare diseases, however, take a disproportionately high share of the health budget, estimated at about 20%. Some of them affect only a few individuals, while others affect hundreds of thousands. Regardless of whether a single rare disease affects millions or just one person, it has enormous medical and social implications, and its impact on the affected individuals and their families is generally devastating. This is especially the case because over 50% of rare diseases affect children, almost half of whom die before the age of 10.

Since 2008, rare diseases have been a priority area for action in EU public health programs (*https://ec.europa.eu/health/ph_threats/non_com/docs/rare_com_en.pdf*). In 2011, the International Rare Diseases Research Consortium (*http://www.irdirc.org/*) was launched, with the ambitious goals of developing diagnostics for most rare diseases and new treatments for 200 of them by 2020.

Historically, rare diseases have become also known as orphan diseases because pharmaceutical companies were not interested in developing treatments for them. However, after much struggle by parents’ non-profit organizations (eg, Families of SMA, Cure SMA, among others), and through the SMA Treatment Acceleration Act, in 2009 SMA entered the list of top 50 medical priorities in the USA. After a series of clinical trials, on December 23, 2016 these joint efforts finally resulted in the approval by the Food and Drug Administration of nusinersen (Spinraza), a medication for all types of SMA. This example shows how a deeper understanding of the genome and disease pathogenesis, combined with energetic involvement of families, may change clinical research and drug development. These efforts could also pave the way for the replacement of larger classifications of common disorders with more precisely defined individual diseases based on genetic markers. Currently, an open-label study is under way to assess the efficacy, safety, tolerability, and pharmacokinetics of multiple doses of nusinersen delivered intrathecally to patients with genetically diagnosed and presymptomatic SMA (*https://clinicaltrials.gov/ct2/show/NCT02386553*), which is estimated to be completed in 2025.

We know that certain disorders, including brain disorders, are inherited. This experiential knowledge has been confirmed by twin studies, but it is only with the emergence of big data and multi-omics studies (genomics, transcriptomics, epigenomics, proteomics, metabolomics, etc) that diagnostic and therapeutic approaches have been reformulated under the umbrella term of personalized medicine. New omics technologies have not only contributed to a better molecular characterization of diseases, especially rare diseases, but have also provided a new level of understanding of the enormous complexity of disease mechanisms (3). To respond to this level of complexity, we will need novel approaches, particularly those that better address the nonprotein coding regions of the genome (4) and its three-dimensional organization (5). Linear hypotheses for any given research proposal or biomedical problem are no longer considered scientifically sound and have been increasingly replaced by machine learning and artificial intelligence (AI) algorithms. Nevertheless, although it is perfectly clear that each patient differently responds to a drug or an intervention on the basis of his or her individual genetic and molecular characteristics, due to the high costs of personalized medicine, all public health systems are still promoting primarily a “one-size fits all” approach. Hopefully, once the challenges of big data integration and translation from basic to clinical research are overcome, innovations based on personalized health care will be increasingly replacing old health care practices.

One of the main obstacles to a full implementation of personalized medicine is that knowledge of genetic underpinnings of a given disorder is insufficient to understand disease pathophysiology, symptomatology, and course. For example, amyotrophic lateral sclerosis (ALS) and frontotemporal dementia (FTD) have for many decades been considered separate diseases. However, some ALS patients had additional symptoms, such as cognitive decline. This was first associated with the *TDP-43* gene, but today we know that TDP-43 neurodegenerative syndrome occurs across a spectrum of conditions from ALS to FTD, whereas the expansion of the hexanucleotide repeat in the *C9ORF72* gene may cause only ALS, only FTD, or combinations of both (6). Therefore, it emerges that one gene can produce a similar clinical picture of both familial or sporadic disease, or may lead to the same syndrome or combination of syndromes. Conversely, multiple genes and pathways may cause the same clinical syndrome of Parkinson’s disease (PD). For example, mutations in *PARK2*, *PARK4,* and *PARK6* genes are linked to mitochondrial dysfunction, whereas Lewy body accumulation can cause dementia (*https://www.medpace.com/rare-diseases-are-showing-the-way-toward-precision-medicine-part-1/*). The finding of an increased risk of PD in individuals with Gaucher’s disease compared with the general population led to the discovery that mutations of *GBA* gene for lysosomal enzyme glucocerebrosidase are foundational in some patients with PD. Patients with* GBA*-associated parkinsonism exhibit varying parkinsonian phenotypes, an earlier age of onset, and more associated cognitive changes than patients with parkinsonism without *GBA* mutations (7).

These examples illustrate how a top-down approach to understand a disease, from signs and symptoms through anatomical and functional substrates to pathways, risk factors, and search for a final common pathway, has dramatically changed to include untargeted omics approaches combined with AI-driven pattern recognition and prediction. Instead of approaching single syndromes by searching for a common final pathway, every individual should be diagnosed and treated as having a separate disease. Such approach has the additional advantage that, based on biomarker findings (imaging, biochemical, or other), it is possible to detect the earliest changes in a prodromal stage and treat a disorder before the clinical onset of symptoms.

In rare diseases, this has become a regular approach because these diseases are driven by a genetic mutation (over 80% of rare diseases have a strong genetic background), which is the obvious treatment target. Beyond SMA treatment, many more drugs for rare diseases were approved in 2016, so that altogether 41% of all drug approvals for that year were for the treatment of rare diseases.

Unlike neurological disorders, and in spite of the strong evidence for heritability, there has been a much slower progress in psychiatric diseases such as schizophrenia and bipolar disorder. One of the main reasons is that genome- and epigenome-wide association studies on these diseases have generally been very inconsistent. Loci involved in these diseases are often single nucleotide polymorphisms, and they do not correlate with a neuropathology, which is far less well-defined. Considering that schizophrenia is associated with over 3000 genes, its molecular basis is even more diverse than its symptomatic diversity. Therefore, the understanding of its pathogenetic mechanisms requires the understanding the action of a large number of genes, including those of the neuroimmune system, their products, and regulatory elements, and those involved in neural crest cells migration and domestication (8), as well as the consequences of their overexpression and/or decreased expression in the context of brain development (9). In other words, the number of genes affecting behavior in schizophrenia is too large for us to make targeted therapies. In addition, intervening in the brain has always been hindered by problems of drug delivery.

Recent breakthroughs in advanced gene and cell therapies have potentially provided the first treatments for some rare diseases. Some of these approvals were highly controversial, as were their huge costs. However, it remains the case that over 90% of rare diseases lack an approved treatment (*https://www.orpha.net/consor/cgi-bin/Drugs_Search.php?lng=EN*). Human induced pluripotent stem cells and genome editing technology, particularly CRISPR/Cas, have been tremendous advances, quickly progressing toward clinical development, although not without ethical issues remaining to be resolved. While most of these developments are very encouraging, much remains to be done. In the years to come, the development of effective, safe, and affordable new treatments for rare diseases will require coordinated involvement of all relevant stakeholders, from patients and their families and associations to other funders, researchers, pharmaceutical companies, clinicians, and governments.
